# Keeping my professional development continuous

**Published:** 2017-05-12

**Authors:** Wanjiku Mathenge

**Affiliations:** 1 Africa Regional Medical Advisor: Fred Hollows Foundation; Consultant Ophthalmologist: RIIO/Dr Agarwal's Eye Hospital, Kigali, Rwanda.

**Keeping up professionally means knowing what is current and then selecting what is applicable to your work. This requires a personal commitment to recognise the need, find the time and seek resources. The process is lifelong and can become part of your routine.**

**Figure:**
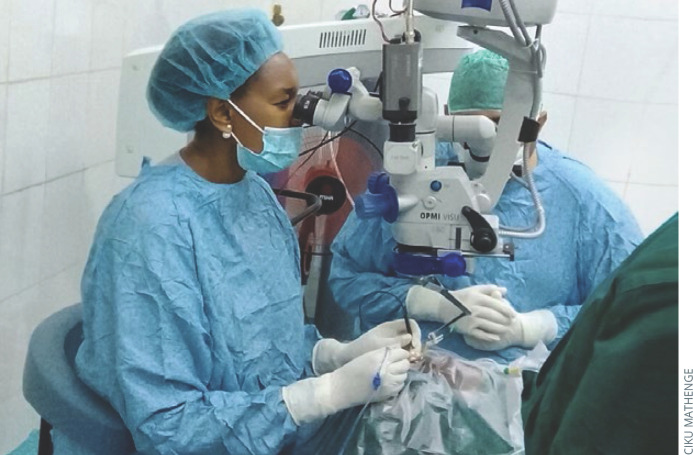
Performing Vitreoretinal surgery under supervision. RWANDA

When I graduated as an ophthalmologist in Africa I really felt I had achieved my goal!; but the reality of working in the field was very different. With each patient, my knowledge and skills were challenged, I found that surprisingly, many patients knew much more than I thought they did.

**Decisions I made as a young ophthalmologist have helped me keep up with my own learning and development**

1. **Formal learning – obtaining CME points.** I decided that once every two years I would save up and attend one major international conference such as the IAPB, WOC or MEACO. I not only attend, I participate by presenting papers or chairing sessions. I receive external sponsorship about 40% of the time. In addition I attend and present at every local annual congress of the College of Ophthalmology of Eastern, Central and Southern Africa (COECSA).
2. **Informal learning – routine reading with no pressure.** I subscribe to ophthalmology journals and newsletters – especially the free online ones. I subscribe to online CPD sites, webinars, and e-CPD sites and download articles that I need to read. I find that when the next patient is sitting in front of me somehow my head will automatically sift through the good and bad articles that I have read, and help me reach a sensible decision.
3. **I keep a network of mentors which has proved invaluable.** I have been inviting these mentors to come and teach me surgery or research in my own set-up with my staff. This helps me and my team to keep up to date with new techniques even after they leave.

Even in a small town like Nakuru patients asked me questions such as “You say you cannot help me here, is there anywhere in the world I can get the help I need? I ‘Googled’ and found…” I soon realised that to answer them honestly I needed to know a great deal more. The intensity and the competitiveness of ophthalmology surprised me too. I was “here” and I needed to be “there”. Being “there” meant staying on top of the game as far as all the pathology, conditions and treatments that I needed to know. The trouble is – “there” is constantly shifting with the advent of new techniques and equipment.

Besides clinical skills there were growing demands relating to management, communications and technology. First recognise your needs and then the path to learning and acquiring the skills can be through a formal or an informal process.

I have found it very useful to be an active member of my local ophthalmology society where I can participate in CPD activities. In Rwanda, my own institute is a registered CPD provider. When CPD became mandatory I found it easier to become more structured in my approach and avoid a last minute scramble for points in order to maintain registration with the Medical Council.

Mandatory CPD is often seen as a “stick,” but it provides thematic guidance and structure that is often harder to organise on your own.

I keep a folder on my laptop called CPD in which I drop all the activities as I do them so that when it comes to submitting to the council, my records are all in one place.

## Conclusion

It is necessary to take ownership and responsibility for your own learning, have formal and informal processes in place and dedicate regular time for reading and keeping professional development continuous.

